# Clinical Utility of Wearable Sensors and Patient-Reported Surveys in Patients With Schizophrenia: Noninterventional, Observational Study

**DOI:** 10.2196/26234

**Published:** 2021-08-09

**Authors:** Adrienne C Lahti, Dai Wang, Huiling Pei, Susan Baker, Vaibhav A Narayan

**Affiliations:** 1 Department of Psychiatry and Behavioral Neurobiology University of Alabama at Birmingham Birmingham, AL United States; 2 Janssen Research & Development Raritan, NJ United States; 3 Janssen Research & Development Titusville, NJ United States

**Keywords:** activity, relapse, schizophrenia, sleep, wearable devices, mobile phone

## Abstract

**Background:**

Relapse in schizophrenia may be preceded by early warning signs of biological, sensory, and clinical status. Early detection of warning signs may facilitate intervention and prevent relapses.

**Objective:**

This study aims to investigate the feasibility of using wearable devices and self-reported technologies to identify symptom exacerbation correlates and relapse in patients with schizophrenia.

**Methods:**

In this observational study, patients with schizophrenia were provided with remote sensing devices to continuously monitor activity (Garmin vivofit) and sleep (Philips Actiwatch), and smartphones were used to record patient-reported outcomes. Clinical assessments of symptoms (Positive and Negative Syndrome Scale and Brief Psychiatric Rating Scale) were performed biweekly, and other clinical scales on symptoms (Clinical Global Impression-Schizophrenia, Calgary Depression Scale), psychosocial functioning, physical activity (Yale Physical Activity Survey), and sleep (Pittsburgh Sleep Quality Index) were assessed every 4 weeks. Patients were observed for 4 months, and correlations between clinical assessments and aggregated device metrics data were assessed using a mixed-effect model. An elastic net model was used to predict the clinical symptoms based on the device features.

**Results:**

Of the 40 patients enrolled, 1 patient relapsed after being stable with evaluable postbaseline data. Weekly patient-reported outcomes were moderately correlated with psychiatric symptoms (Brief Psychiatric Rating Scale total score, *r*=0.29; Calgary Depression Scale total score, *r*=0.37; and Positive and Negative Syndrome Scale total score, *r*=0.3). In the elastic net model, sleep and activity features derived from Philips Actigraph and Garmin vivofit were predictive of the sitting index of the Yale Physical Activity Survey and sleep duration component of the Pittsburgh Sleep Quality Index. On the basis of the combined patient data, a high percentage of data coverage and compliance (>80%) was observed for each device.

**Conclusions:**

This study demonstrated that wearable devices and smartphones could be effectively deployed and potentially used to monitor patients with schizophrenia. Furthermore, metrics-based prediction models can assist in detecting earlier signs of symptom changes. The operational learnings from this study may provide insights to conduct future studies.

**Trial Registration:**

ClinicalTrials.gov NCT02224430; https://www.clinicaltrials.gov/ct2/show/NCT02224430

## Introduction

### Background

Psychotic symptom exacerbation and relapse are frequently observed in patients with schizophrenia and can lead to a decline in social functioning, reduced treatment response, and worsening of clinical outcomes [1]. These patients and their caregivers experience an increased burden because of relapse and consequent hospitalization [2-4]. Relapse in schizophrenia may be preceded by early warning signs, including depressed mood; social withdrawal; and changes in physical activities, feelings, emotions, and sleep disturbances [5]. Therefore, identifying warning signs can enable early intervention to avoid subsequent relapse events [6]. Symptom onset can be rapid; however, continuous monitoring may provide an advantage for early intervention [7].

Web-based data capturing technologies such as Information Technology–Aided Relapse Prevention in Schizophrenia have been piloted to recognize warning signs based on patient reporting of prodromal symptoms of relapse [8]. However, the frequency at which it is practical to obtain this information and the subjective nature of patients’ and caregivers’ responses pose challenges. Small, unobtrusive remote sensing devices, along with existing mobile technologies, make it possible to capture real-time data on patients’ activities, sleep patterns, behaviors, and symptoms. More recent studies have indicated the general availability and acceptability of devices for remote assessment and management [9-11]. Smartphones are commonly used and have multiple embedded sensors (eg, accelerometer, microphone, GPS, and camera). These can be leveraged to collect symptom reports through patient-reported outcome (PRO) surveys and to collect passive data to measure changes in behavior [9,12-19]. A consumer wrist-worn smartwatch or fitness band can additionally provide measurements of precise and objective activity patterns spanning sleep-rest and active-awake periods. These devices have an advantage of generating continuous streaming data that are more reproducible and less obtrusive than relying on patient and caregiver reports alone. In addition, changes in device compliance may itself be a signal and indicate a clinically relevant change in behavior [9,17].

### Study Objectives

This clinical study was designed to explore the signatures of relapse. However, because patients were mainly recruited from outpatient clinics and followed up for 4 months—a short period to observe relapses in stabilized patients—there were insufficient relapses to perform the primary objective. We subsequently evaluated the feasibility of using wearable devices (singly and in combination) and self-reporting technologies to identify potentially predictive symptom correlates in patients with schizophrenia or schizoaffective disorder who are at increased risk of relapse. Continuous monitoring using wearable devices (eg, fitness bands and smartwatches) and self-reporting via smartphones were used in this study, and predictive modeling was applied to examine the correlations between clinical assessments and aggregated metrics data.

## Methods

### Overview

This study was conducted at the University of Alabama, Birmingham (UAB), from August 8, 2015, to March 28, 2016. The protocol was approved by the UAB Institutional Review Board, and all patients or legally authorized representatives provided written informed consent and Health Insurance Portability and Accountability Act authorization before the start of the study.

### Patients

Men and women (aged ≥19 years) who met the Diagnostic and Statistical Manual of Mental Disorders, Fifth Edition (DSM-5), criteria for schizophrenia or schizoaffective disorder diagnosed based on the Structured Clinical Interview for DSM-5-Text Revision Axis I Disorders using the Diagnostic Interview for Genetic Studies-4.0 were included in this study. The target population included patients discharged from the inpatient psychiatry unit, emergency department, or outpatient clinics of UAB who were maintained on a stable dose of antipsychotic medication that remained unchanged for 2 weeks before the start of the study.

Patients were excluded if they had physical or clinical disabilities or both, such as hearing, vision, or motor impairment, leading to difficulties in operating a smartphone or responding to prompts (determined using a demonstration smartphone for screening); severe substance use disorder (≥6 symptoms) according to DSM-5 Level 2–Substance Use–Adult scale (adapted from the National Institute on Drug Abuse-Modified Alcohol, Smoking, and Substance Involvement Screening Test); or if they were enrolled or planning to enroll in an interventional study for the treatment or prevention of worsening of symptoms of schizophrenia.

### Study Design

**Figure 1:**
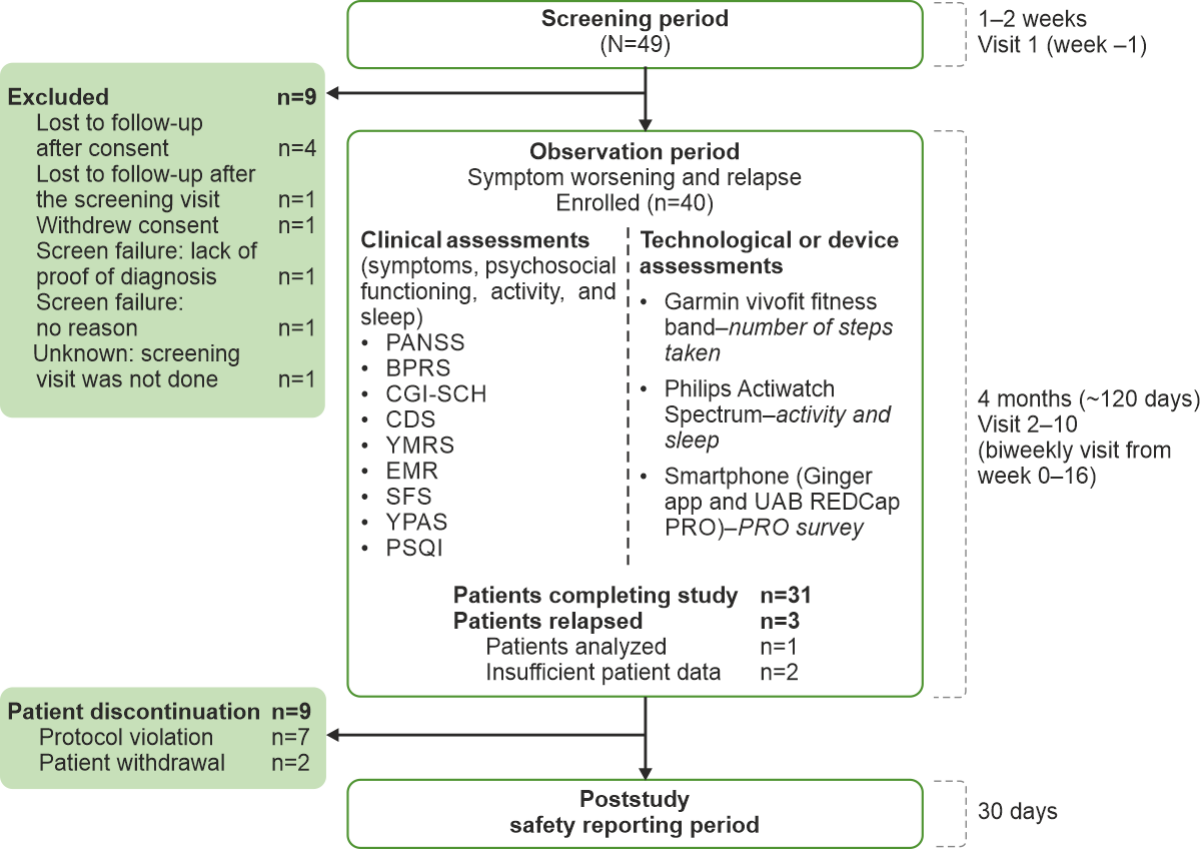
Study design and patient disposition. Patient-reported outcome self-reported symptom questionnaire administered every other day (bidaily) or weekly. Patients were allowed to continue their usual standard of care and antipsychotic treatment as prescribed by their physician but maintained a stable dose, which had not changed for 2 weeks before enrollment. BPRS: modified Brief Psychiatric Rating Scale; CDS: Calgary Depression Scale; CGI-SCH: Clinical Global Impression-Schizophrenia; EMR: electronic medical record; PANSS: Positive and Negative Syndrome Scale; PRO: patient-reported outcome; PSQI: Pittsburgh Sleep Quality Index; REDCap: Research Electronic Data Capture; SFS: Social Functioning Scale; UAB: University of Alabama, Birmingham; YMRS: Young Mania Rating Scale; YPAS: Yale Physical Activity Survey.

This was a noninterventional, observational, exploratory clinical study in which patients were allowed to continue with their usual standard of care and antipsychotic treatment as prescribed by their physician. Patients were screened for eligibility for up to 2 weeks. Enrolled patients were observed for relapse for 4 months (approximately 120 days; observation or study participation period), followed by a 30-day poststudy safety reporting period (Figure 1). Patients were considered to have experienced relapse if they had a rating of moderately severe, very severe, or extremely severe (item score ≥5)—in the previous 2 weeks—for ≥1 item on the Positive and Negative Syndrome Scale (PANSS) positive subscale (items P1-P7) or ≥2 items on the negative subscale (items N1-N7) [20] or if they had symptom exacerbation (increased PANSS total score) that required a change in antipsychotic medication or upward dosage adjustment.

Wearable remote sensing devices and a smartphone were provided to eligible patients for use during the observation period. All patients had to undergo a training tutorial for the devices and smartphone based on their individual learning needs. Repeated practice was performed until patients were comfortable using these items. The importance of refraining from tampering or attempting to deactivate the devices was conveyed to the patients. The confidence of patients with using the devices was assessed, and retraining was performed, if required. Potential predictors of symptom worsening or relapse (eg, sleep quality) were collected using remote sensing devices, and the results were subsequently compared with standard clinical assessments. Patients did not have access to the activity and sleep data generated by the wearable devices, so that behavior was independent of feedback.

The 30-day poststudy safety reporting period was per Janssen Adverse Event Reporting Requirements for Noninterventional Studies, wherein all adverse events were recorded from the first use of the Janssen products and for 30 days after the last use of these Janssen drug products within the study.

### Clinical Assessments

During the observation period, clinical assessments were performed for symptom worsening and relapse identification. PANSS and modified Brief Psychiatric Rating Scale (BPRS) [21] were assessed every 2 weeks (biweekly), whereas Clinical Global Impression-Schizophrenia (CGI-SCH) [22], Calgary Depression Scale (CDS) [23], Young Mania Rating Scale [24], and electronic medical records were assessed every 4 weeks. Physical activity was assessed using the Yale Physical Activity Survey (YPAS) [25], and sleep was monitored using the Pittsburgh Sleep Quality Index (PSQI) [26] every 4 weeks. Physicians completed the CGI-SCH [22] severity scale for each patient, and a trained study coordinator administered the PANSS to patients. Clinical assessments performed at baseline for patient characterization included psychosocial functioning (assessed using the Social Functioning Scale [27]) or quality of life, cognitive functioning, impulsivity, and measure of addiction. Patient self-reports were also used to assess symptom status and relapse.

### Device Data Collection and Processing

#### General Information

The study was monitored according to the sponsor’s current standard operating procedures for the monitoring of clinical trials, and activities were implemented to ensure proper operational study oversight. These activities focused on identifying and resolving operational and quality issues to ensure data integrity, protocol compliance, and safety of the study participants. Written instructions were provided for collecting source documentation, which was reviewed for accuracy and completeness by the sponsor during on-site monitoring visits and underwent internal data reviews throughout the study and at the time of database lock. Discrepancies were resolved with the investigator or designees, as appropriate.

The UAB clinical site captured all clinical assessments using source documents, and these data were entered into the REDCap (Research Electronic Data Capture) system. The nature and location of all source documents were identified to ensure that all sources of original data required to complete data collection were known to the sponsor or investigator and study site personnel.

Smartwatches or fitness bands can monitor activity based on the time frame, duration, and intensity of movement. The unprocessed data collected from wearable devices (eg, accelerometry) may not directly represent variables or metrics that are amenable to patient-relevant interpretation or traditional prespecified statistical measures. The raw data are reduced by the device manufacturers to metrics representing the average state of an individual during specified periods (eg, steps taken, time spent resting or sleeping, and activity intensity levels). These behavioral and lifestyle measures provided a set of metrics that were tested independently and collectively as a pattern toward the specified aims. Analytics can be derived from the patterns that are normal (baseline) for a given patient to detect any relevant changes during clinical follow-up. For sleep, domains that could be assessed through remote sensing devices include onset time, duration, and quality; frequency and pattern of sleep disruptions can be monitored by the number and duration of movements. Similarly for activity, steps per day (mobility) and patterns of daily activity—distribution of high-, medium-, and low-intensity activities—can be assessed. Mobile phone reporting of clinically relevant metrics such as medication compliance; well-being; and the degree of symptom experience, such as seeing or hearing things, could also be assessed.

Device data and PRO responses collected between clinical visits were aggregated to test correlations with the clinical visits and outcomes. Compliance using the devices was also monitored (defined as using or wearing either all devices for 50% of the time in 24 hours or 2 of 4 devices during the 4-month observation period).

#### Garmin Vivofit Fitness Band

Garmin vivofit is a wristband with an easy-to-read display that was worn at all times by patients to track ambulatory activity (number of steps taken every 15 minutes). A single device was dispensed at visit 1 with instructions for use. At every visit to the clinic, data from the device were downloaded onto the site computer and stored in a secure location. The Garmin vivofit device is a consumer-grade fitness device. Consumer devices have the benefit of higher user acceptance as a social norm. The disadvantage is that the measurements are not clinically validated, and specific firmware versions need to be tracked, as they can impact the results. Despite these challenges, if the intended outcome is detecting relative changes in behavior for individuals rather than cross-sectional studies, they can provide some utility.

#### Philips Actiwatch Spectrum

The Philips Actiwatch is a wristwatch with an actigraphy system for tracking objective data on off-wrist status, sleep-wake, activity count, and light exposure. Data were collected in 30-second epochs. This actiwatch was worn 24×7, is designed for clinical trials and populations, and is well established for actigraphy-based sleep assessments [28]. A single wristwatch was dispensed to patients at visit 1. At every visit to the clinic, data from the device were downloaded from a proprietary docking station supplied by Philips.

As per the study protocol, patients were requested to wear both Philips Actiwatch and Garmin vivofit devices at all times without additional guidance on the arm preference.

#### Smartphone

Smartphone apps were used in the study to collect PROs consisting of self-assessment and symptom-tracking questionnaires. One set of questions was given every 2 days (bidaily), and the other set of questions was given weekly throughout the observation period. The Ginger app was used at the beginning of the study for collection, but patients were migrated to the UAB REDCap PRO survey system. The REDCap system collected the same information at the same frequency as the originally deployed Ginger app but had the advantage of direct capture in the clinical database rather than needing to capture the data periodically through a third-party upload. The reason for the switch was that the duration of the trial recruitment period exceeded that of the contracted services with Ginger. A smooth transition was easily developed and implemented, and no data were lost.

Patients who had personal smartphones were asked to download the Ginger app. The site provided a smartphone if the patient did not own one. Patients were restricted to the use of only one personal smartphone with the Ginger app during the course of the study. To prevent the erroneous collection of nonpatient data, the use of the smartphone was restricted to the patient only and was not to be shared with others.

In addition, patients were instructed on the completion of UAB REDCap PRO surveys via an email set up for each patient by the UAB. The email contained a link for access to the survey. Responses to the survey were collected within REDCap through a secure web-based app module to manage and build the web-based surveys.

Both Ginger and REDCap data were recorded on the Health Insurance Portability and Accountability Act–compliant UAB server. At the end of the study, all patients were asked to return their wristwatches. Patients completing the study were allowed to keep the site-issued smartphone, but the monthly plan was terminated. Patients did not have access to the data generated during the course of the study.

### Analytical Methods

#### Statistical Analysis

The sample size for the study was estimated based on previous experience, assuming that 35% to 40% of patients may experience a full significant exacerbation of psychotic symptoms or relapse during the course of the study. The level of relapse anticipated within 120 days for patients treated for a current relapse was based on the experience of UAB in their clinics. For patient characteristics, all continuous variables were summarized using descriptive statistics, and the categorical variables were summarized using frequency measures. As the analyses were exploratory in nature, a two-sided significance level of 5% was used, unless specified otherwise, for all statistical tests. Multiplicity adjustments were not made for the analysis.

Without significant clinical changes to detect or model, we assessed the within-patient stability of the clinical scales using the intraclass correlation coefficient (ICC), a metric commonly used in psychometrics to assess the test-retest and interrater reliability. If the patient-level variance is small for stable patients, it is easier to detect potentially important deviations from individualized norms. The ICC was calculated using a mixed-effect model, in which patient was included as a random effect.

Table 1 summarizes all the metrics obtained from the devices. These metrics were aggregated biweekly to generate the feature sets for predictive modeling. Means and SDs of the device metrics during the 2 weeks immediately before the corresponding clinical assessment were calculated. The summary statistics of all device metrics were combined as the feature set for models predictive of clinical assessments.

Associations among the clinical symptoms and between clinical symptoms and device variables were assessed using mixed-effect models. For each pair, one variable was considered as the response variable and the other variable was considered as the independent variable. Patient was included in the model as a random effect to account for correlations among the repeated measures. Each data variable was scaled so that each had a variance of 1. Testing whether the dependent and independent variables are correlated is equivalent to testing whether the coefficient of the independent variable is 0.

Elastic net [29] was applied to build models using linear regression to predict the clinical assessments and the patient-reported activity and sleep scales using feature sets constructed from the device data. Ten-fold repeated cross-validation with 30 repeats was also applied to train the models and assess the performance of the model. The repeated cross-validation was conducted at the patient level, where data on 10% of patients were held out for validation. The performance of the predictive models was assessed using the root mean square error and the R^2^. The R^2^ was calculated as the difference between the total sum of squares and the sum of squares owing to the error sum of squares divided by the total sum of squares. When the R^2^ is calculated based on cross-validation results and the sum of squares owing to error is calculated based on out-of-sample predictions, the estimated R^2^ may be negative.

**Table 1:** Metrics obtained from Philips Actigraph, Garmin vivofit, and surveys.

Device and metric			Frequency
**Philips Actigraph**			
	Time spent sedentary (minutes)	Daily	
	Time spent on low-intensity activities (minutes)	Daily	
	Time spent on moderate-intensity activities (minutes)	Daily	
	Time spent on vigorous-intensity activities (minutes)	Daily	
	Total activity count	Daily, nightly	
	Average activity count	Daily, nightly	
	Maximum activity count	Daily, nightly	
	Duration of active or sleeping (minutes)	Daily, nightly	
	Percentage of time with invalid sleep-wake status	Daily, nightly	
	Time spent awake (minutes)	Daily, nightly	
	Percentage of time spent awake	Daily, nightly	
	Number of wake bouts	Nightly	
	Average duration of wake bouts (minutes)	Nightly	
	Time spent sleeping (minutes)	Daily, nightly	
	Percentage of time spent sleeping	Daily, nightly	
	Sleep onset latency (minutes)	Nightly	
	Time spent resting after waking up (minutes)	Nightly	
	Time of valid rest (minutes)	Nightly	
	Sleep efficiency	Nightly	
	Sleep start time (hours:minutes)	Nightly	
	Rest start time (hours:minutes)	Nightly	
	Sleep fragmentation	Nightly	
**Garmin** **vivofit**			
	Time spent sedentary (seconds)	Daily	
	Time spent walking (seconds)	Daily	
	Total steps	Daily	
	Number of epochs	Daily	
	Number of steps during nighttime	Nightly	
**Survey**			
	Bidaily survey summary score	Bidaily	
	Feel down or depressed	Bidaily	
	Feel confused or have trouble with your thinking	Bidaily	
	Feel stressed or overwhelmed	Bidaily	
	See or hear things that other people could not see or hear	Bidaily	
	Feel suspicious or paranoid	Bidaily	
	Have trouble sleeping the night before	Bidaily	
	Weekly survey summary score	Weekly	
	Feel anxious or nervous	Weekly	
	Feel unmotivated	Weekly	
	Have trouble getting things done	Weekly	
	Missed any schizophrenia medications in the past 7 days	Weekly	

#### Detecting Statistically Relevant Change in Streaming Data

**Figure 2:**
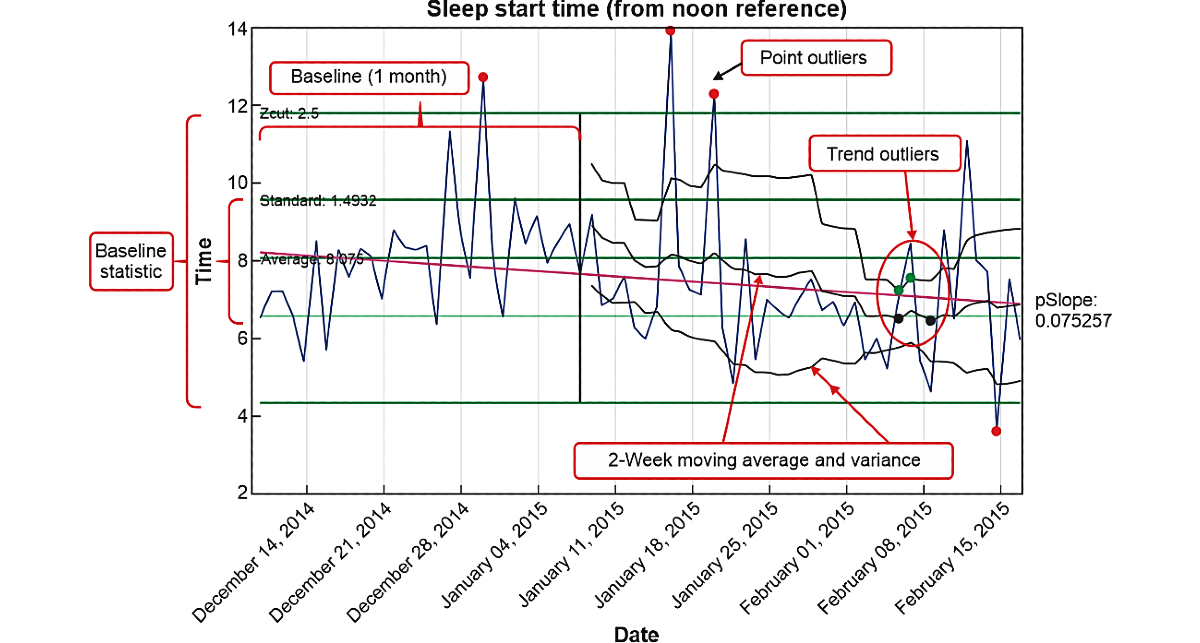
Sleep onset time for a single relapse patient. Outliers and trends may have causal explanations other than relapse but allow an opportunity to have conversations about behaviors with objective data. The first month was used to establish baseline normal data. Outlier alerts can be set based on baseline mean and variance. In this case, outlier detection alerts were set at 2.5 SDs from the baseline population distribution. After the baseline, a moving average window of 2 weeks was calculated and overlayed on daily data and was used to look for significant changes or trends from the baseline mean or variance. Finally, statistically relevant changes in the slope over a defined interval can indicate relevant changes. The flagged points represent outliers or changes above a set threshold. Flags noted on the moving average mean indicate significant differences for the moving average mean, and flags noted on the moving average variance indicate significant differences in variance from the baseline. For example, examining the sleep onset data, there is a significant trend toward earlier sleep times as the patient approached relapse. The average sleep onset time at baseline was 8 PM. Near relapse, the data had trended to a 2-week average around 6:30 PM.

There is evidence that detectable signs of relapse may be specific to an individual [17]. As such, it is important to develop metrics that can detect statistically relevant changes based on a person’s own data rather than what may be normal for individuals. Metrics derived from the devices or PRO can be monitored as a *real-time* streaming process similar to process control in manufacturing or even the stock market. A stable longitudinal period can be used to establish a baseline norm (mean and variance) for each metric of interest. At any subsequent point, statistical tests can be performed for point outlier status of the daily tracked measures or if the short-term mean and variance of a defined measurement period are statistically different from the baseline measures, indicating that a trend may be occurring. Outliers and trends may have causal explanations other than relapse; however, detected changes create an opportunity to have conversations with the patient about behaviors, with objective data. For example, in Figure 2, examination of sleep onset data revealed a significant trend toward earlier sleep times as the patient approached relapse. The average sleep onset time at baseline was 8 PM. Near relapse, the data trended to a 2-week average around 6:30 PM.

It is relatively easy to detect statistically relevant changes in the streaming metrics. The challenge is assigning causality to the change without an understanding of the context [13,16,17]. Most detected changes are probably not relevant to relapse but could be used in a clinical setting for follow-up or conversations with caregivers based on these objective data.

## Results

### Patient Disposition and Baseline Characteristics

A total of 49 patients were screened, and 40 patients were enrolled in the study. In total, 9 participants were screened out because of loss to follow-up after consent (n=4) and after the screening visit (n=1), consent withdrawal (n=1), screen failure—lack of proof of diagnosis (n=1) and no reason (n=1), and unknown—screening visit not being performed (n=1).

Of these 40 patients, 31 (78%) completed the study and 9 (23%) discontinued the study (protocol violations: 18%, 7/40 and withdrawal of consent: 5%, 2/40; Figure 1). Only 8% (3/40) of patients experienced a relapse during the study, of which only 1 patient had sufficient postscreening data to establish a reference baseline. The enrolled patients had a higher proportion of men (25/40; 63%), a median age of 40.3 years, and a mean baseline BMI of 34.9 kg/m^2^. Most patients (38/40;95%) were on at least one antipsychotic medication during the course of the study, and the most common medication was Risperdal Consta (28/40; 70%). All patients continued their prescribed medication throughout the study (Table 2).

**Table 2:** Patients’ demographics and baseline characteristics (N=40).

Characteristic			Values
Age (years), median (range)			40.3 (19-65)
**Gender, n (%)**			
	Women	15 (38)	
	Men	25 (63)	
**Race, n (%)**			
	Black or African American	29 (73)	
	White	11 (28)	
Baseline BMI (kg/m^2^), mean (SD)			34.9 (8.65)
Baseline PANSS^a^ total score, mean (SD)			35.7 (5.02)
**Baseline CGI-S^b^ score, n (%)**			
	Normal, not at all ill	1 (3)	
	Borderline mentally ill	4 (10)	
	Mildly ill	11 (28)	
	Moderately ill	17 (43)	
	Markedly ill	7 (18)	

^a^PANSS: Positive and Negative Syndrome Scale.

^b^CGI-S: Clinical Global Impression-Severity scale.

### Clinical Assessments

For the patient experiencing a relapse, the CGI disease severity scores worsened from 2 to 5 (borderline mentally to markedly ill), and the global improvement changed from 4 to 6 (*no change* to *much worse*). The PANSS domains that worsened at relapse included positive subscale items (delusions-P1 and conceptual disorganization-P2), negative subscale items (poor rapport-N3), and general psychopathology scale items (somatic concern-G1, anxiety-G2, tension-G4, depression-G6, uncooperative-G8, lack of judgment-G12, and disturbance of volition-G13). The BPRS scale worsened in the subdomains of somatic concern, anxiety, conceptual disorganization, tension, depressive mood, uncooperative, and disorientation. The Young Mania Rating Scale worsened in the subdomains of sleep, language, thought disorder, and insight, and the CDS worsened in the subdomains of depression and early wakening.

### Correlations Among Clinical Symptoms

For the 40 enrolled patients, the within-patient stability of the clinical symptom scales as assessed by ICC showed either excellent or good agreement, suggesting that most of the assessed symptoms were stable throughout the study (Multimedia Appendix 1). When assessing the association among clinical symptoms, the PANSS total score and the BPRS total scores showed a very strong correlation (*r*=0.97). However, the Clinical Global Impression-Severity scale score did not strongly correlate with the PANSS and BPRS scores (Table 3).

**Table 3:** Associations among psychiatric symptoms (N=40).

Symptom scores		BPRS^a^ total score	CGI-S^b^ score	CDS^c^ total score	YMRS^d^ total score
**PANSS^e^ total score**					
	Coefficient	*0.97^f^*	0.12	*0.68*	*0.29*
	*P* value	*<.001*	.09	*<.001*	*.03*
**BPRS total score**					
	Coefficient	—^g^	*0.15*	*0.65*	*0.34*
	*P* value	—	*.01*	*<.001*	*.002*
**CGI-S score**					
	Coefficient	—	—	*0.24*	0.23
	*P* value	—	—	*.003*	.18
**CDS total score**					
	Coefficient	—	—	—	*0.44*
	*P* value	—	—	—	*.003*

^a^BPRS: modified Brief Psychiatric Rating Scale.

^b^CGI-S: Clinical Global Impression-Severity scale.

^c^CDS: Calgary Depression Scale.

^d^YMRS: Young Mania Rating Scale.

^e^PANSS: Positive and Negative Syndrome Scale.

^f^Italic values indicate significant correlation (*P*<.05).

^g^Not applicable.

### Device-Based Assessments

With 1 patient having a stable baseline before relapse, it is not possible to infer statistical indicators from the data that might have suggested an impending potential relapse. However, a few anecdotal observations have been made. Before relapse, the day-to-day variation in mobility was very high, ranging from 1 to 7 miles per day, as captured by the Garmin vivofit fitness band. Some disrupted sleeping was captured using the Philips Actiwatch. The patient reported a spike in *the feeling of suspicion* and missed his medication dose at this time.

### Data Coverage and Patient Compliance

Across all patients, the range of days each device was in use provided the maximum number of observable days for each patient-device combination. On the basis of the combined patient data, a high percentage of data coverage and compliance was observed for each device. Using the Garmin vivofit, Philips Actiwatch Spectrum, PRO bidaily survey, and PRO weekly survey, the data coverage was 96%, 92%, 80%, and 89%, respectively, and the device compliance was 97%, 94%, 82%, and 88%, respectively.

### Correlations Between Clinical Symptoms and Device Metrics

The low variability in clinical symptoms prevented assessments of individual-based changes in device data with clinical status changes. Subsequently, an analysis was performed to directly correlate the aggregated device metrics with related clinical measures. Correlations were observed between the Philips Actigraph sleep features and the PSQI sleep duration component. Features such as sleep duration, time spent sleeping, and time of valid rest significantly correlated with PSQI sleep duration (*r*=0.36, *r*=0.36, and *r*=0.34, respectively). Sleep start time and resting-state time showed more modest but significant correlations with PSQI sleep disturbances (*r*=−0.26 and *r*=−0.25, respectively; Table 4).

Correlations were observed between the Philips Actigraph activity features and the YPAS individual indices (Table 5). Garmin activity feature—total steps taken—showed a modest correlation with the BPRS total score (*r*=−0.23; *P*=.03). Garmin activity metrics also showed correlations with the YPAS global or individual indices (Tables 6 and 7).

**Table 4:** Associations between Philips Actigraph sleep features and the Pittsburgh Sleep Quality Index global and component scores.

Metric	PSQI^a^ sleep duration		PSQI sleep disturbances	
	Coefficient	*P* value	Coefficient	*P* value
Total activity count	0.03	.80	0.24	.10
Average activity count	−0.14	.22	0.27	.08
Maximum activity count	0	.99	0.17	.23
Duration (minutes)	*0.36^b^*	*<.001*	−0.02	.88
Time spent awake (minutes)	0.11	.30	0.21	.10
Percentage of time spent awake	−0.1	.40	0.25	.09
Number of wake bouts	0.26	.01	0.09	.45
Average number of wake bouts	−0.18	.08	0.22	.11
Time spent sleeping	*0.36*	*.001*	−0.07	.58
Percentage of time spent sleeping	0.1	.40	−0.25	.09
Sleep onset latency (minutes)	0.05	.56	−0.02	.87
Time spent resting after waking up (minutes)	−0.09	.39	nc^c^	nc
Time of valid rest (minutes)	*0.34*	*<.001*	−0.01	.92
Sleep efficiency	0.21	.09	−0.23	.11
Sleep start time (hours:minutes)	−0.01	.93	−*0.26*	*.01*
Rest start time (hours:minutes)	−0.03	.78	−*0.25*	*.01*
Sleep fragmentation	0	.99	0.17	.18

^a^PSQI: Pittsburgh Sleep Quality Index.

^b^Italic values indicate significant correlation (*P*<.05).

^c^nc: the model did not converge.

**Table 5:** Associations between Philips Actigraph activity features and the Yale Physical Activity Survey global or individual indexes.

Activity features		YPAS^a^ global index	YPAS vigorous activity index	YPAS leisure walking index	YPAS moving index	YPAS standing index	YPAS sitting index
**Time spent sedentary (minutes)**							
	Coefficient	−*0.35*^b^	nc^c^	‒0.01	−*0.25*	−*0.36*	*0.27*
	*P* value	*.01*	—^d^	.95	*.02*	*.01*	*.01*
**Time spent on low-intensity activities (minutes)**							
	Coefficient	*0.40*	nc	0.07	*0.26*	*0.40*	−*0.3*
	*P* value	*.003*	—	.61	*.03*	*.003*	*.004*
**Time spent on moderate-intensity activities (minutes)**							
	Coefficient	0.82	0.93	nc	nc	1.03	−0.05
	*P* value	.25	.17	—	—	.26	.97
**Time spent on vigorous-intensity activities (minutes)**							
	Coefficient	0.22	0.29	0.05	nc	0.45	nc
	*P* value	.61	.70	.89	—	.31	—
**Total activity count**							
	Coefficient	*0.33*	nc	nc	0.22	*0.33*	−0.2
	*P* value	*.02*	—	—	.14	*.02*	.06
**Average activity count**							
	Coefficient	0.33	nc	0.06	0.25	*0.33*	−0.22
	*P* value	.06	—	.63	.27	*.03*	.13
**Maximum activity count**							
	Coefficient	0.25	nc	−0.02	0.12	*0.26*	−0.01
	*P* value	.14	—	.89	.25	*.03*	.95
**Duration (minutes)**							
	Coefficient	0.06	0.1	−0.03	0	0.07	0.02
	*P* value	.46	.30	.70	.97	.48	.86
**Time spent awake (minutes)**							
	Coefficient	nc	nc	−0.06	0.11	0.05	0.01
	*P* value	—	—	.57	.26	.81	.91
**Time spent awake (%)**							
	Coefficient	nc	nc	−0.01	0.19	0.06	0.01
	*P* value	—	—	.93	.13	.77	.96
**Time spent sleeping**							
	Coefficient	nc	−0.13	0.01	−0.18	−0.06	0.04
	*P* value	—	.49	.95	.13	.78	.70
**Time spent sleeping (%)**							
	Coefficient	nc	nc	0.01	−0.19	−0.06	−0.01
	*P* value	—	—	.93	.13	.77	.96

^a^YPAS: Yale Physical Activity Survey.

^b^Italic values indicate significant correlation (*P*<.05).

^c^nc: the model did not converge.

^d^Not applicable.

**Table 6:** Associations between Garmin activity features and clinical scores.

Activity features		PANSS^a^ total score	BPRS^b^ total score	CGI-SCH^c^ score	CDS^d^ total score	YMRS^e^ total score
**Time spent sedentary (seconds)**						
	Coefficient	0.15	0.12	0.25	−0.03	nc^f^
	*P* value	.66	.59	.27	.88	—^g^
**Time spent walking (seconds)**						
	Coefficient	−0.15	−0.12	−0.25	0.03	nc
	*P* value	.65	.58	.26	.88	—
**Total steps**						
	Coefficient	−0.21	−*0.23*^h^	−0.07	nc	−0.13
	*P* value	.19	*.03*	.44	—	.40

^a^PANSS: Positive and Negative Syndrome Scale.

^b^BPRS: modified Brief Psychiatric Rating Scale.

^c^CGI-SCH: Clinical Global Impression-Schizophrenia.

^d^CDS: Calgary Depression Scale.

^e^YMRS: Young Mania Rating Scale.

^f^nc: the model did not converge.

^g^Not applicable.

^h^Italic values indicate significant correlation (*P<*.05).

**Table 7:** Associations between Garmin activity features and the Yale Physical Activity Survey global or individual indexes.

Activity features		YPAS^a^ global index	YPAS vigorous activity index	YPAS leisure walking index	YPAS moving index	YPAS standing index	YPAS sitting index
**Time spent sedentary (seconds)**							
	Coefficient	−0.15	−0.14	0.09	−*0.3*^b^	−*0.29*	*0.25*
	*P* value	.21	.26	.59	*.04*	*.03*	*.05*
**Time spent walking (seconds)**							
	Coefficient	0.15	0.14	−0.1	*0.31*	*0.29*	−0.25
	*P* value	.20	.26	.57	*.03*	*.03*	.05
**Total steps**							
	Coefficient	*0.17*	0.1	*0.38*	0.26	0.13	−*0.21*
	*P* value	*.05*	.26	*.03*	.11	.33	*.04*

^a^YPAS: Yale Physical Activity Survey.

^b^Italic values indicate significant correlation (*P*<.05).

A significant but moderate association between survey data features and psychiatric symptoms was observed. The bidaily survey summary score correlated with the BPRS total score (*r*=0.23; *P*=.05) and the CDS total score (*r*=0.37; *P*=.01). The weekly survey summary score was also associated with the PANSS total score (*r*=0.30; *P*=.03), the BPRS total score (*r*=0.29; *P*=.01), and the CDS total score (*r*=0.37; *P*=.01). Specific queries such as *feel down or depressed*, *feel confused or have trouble with your thinking*, *feel stressed or overwhelmed*, and *have trouble getting things done* correlated with the CDS total score (Table 8).

In the elastic net model, survey data (bidaily, weekly, and bidaily+weekly) were observed to be predictive of the PANSS total score, the BPRS total score, and the CDS total score (Table 9). The features derived from the survey data were found to be predictive of the total score, subjective sleep quality component, and sleep disturbance component of the PSQI (Table 4). The features from Philips Actigraph data were also found to be predictive of the sleep latency component of the PSQI. The sleep and activity features derived from the Philips Actigraph and Garmin activity data were observed to be predictors of the sleep duration component of the PSQI (Table 10) and the sitting index of the YPAS (Multimedia Appendix 2).

**Table 8:** Associations between survey data features and psychiatric symptoms.

Survey data features		PANSS^a^ total score	BPRS^b^ total score	CGI-SCH^c^ score	CDSS^d^ total score	YMRS^e^ total score
**Bidaily survey summary score**						
	Coefficient	0.27	*0.23* ^f^	nc^g^	*0.37*	nc
	*P* value	.09	*.05*	—^h^	*.01*	—
**Feel down or depressed**						
	Coefficient	0.28	0.2	nc	*0.34*	0.01
	*P* value	.11	.10	—	*.01*	.93
**Feel confused or have trouble with your thinking**						
	Coefficient	0.25	0.19	nc	*0.35*	0.12
	*P* value	.09	.08	—	*.01*	.34
**Feel stressed or overwhelmed**						
	Coefficient	0.29	0.16	–0.04	*0.25*	0.02
	*P* value	.06	.11	.49	*.04*	.86
**See or hear things that other people could not see or hear**						
	Coefficient	0.26	0.2	0	0.11	nc
	*P* value	.22	.13	.99	.42	—
**Have trouble sleeping the night before**						
	Coefficient	0.10	0.10	–0.07	0.02	–0.04
	*P* value	.39	.22	.48	.84	.69
**Weekly survey summary score**						
	Coefficient	*0.30*	*0.29*	–0.2	*0.37*	0.09
	*P* value	*.03*	*.01*	.26	*.01*	.47
**Feel anxious or nervous**						
	Coefficient	0.09	0.18	–0.17	0.26	0.09
	*P* value	.71	.12	.16	.06	.55
**Feel unmotivated**						
	Coefficient	0.19	*0.23*	–0.06	0.26	0.1
	*P* value	.16	*.04*	.70	.20	.47
**Have trouble getting things done**						
	Coefficient	*0.35*	*0.25*	0.05	*0.28*	0.09
	*P* value	*.04*	*.04*	.73	*.04*	.40
**Missed any schizophrenia medications in past 7 days**						
	Coefficient	0.23	0.03	nc	0.11	0.37
	*P* value	.46	.86	—	.19	.07

^a^PANSS: Positive and Negative Syndrome Scale.

^b^BPRS: modified Brief Psychiatric Rating Scale.

^c^CGI-SCH: Clinical Global Impression-Schizophrenia.

^d^CDS: Calgary Depression Scale.

^e^YMRS: Young Mania Rating Scale.

^f^Italic values indicate significant correlation (*P*<.05).

^g^nc: the model did not converge.

^h^Not applicable.

**Table 9:** Performance of the elastic net models for predicting clinical scores (N=40).

Feature set			RMSE^a^		SD		R^2^		SD
**PANSS^b^ total score**									
	Survey bidaily	*4.27^c^*		*0.03*		*0.11*		*0.01*	
	Survey weekly	*4.23*		*0.05*		*0.12*		*0.02*	
	Survey (bidaily+weekly)	*4.20*		*0.04*		*0.13*		*0.02*	
**BPRS^d^ total score**									
	Survey bidaily	*3.90*		*0.03*		*0.19*		*0.01*	
	Survey weekly	*3.94*		*0.06*		*0.17*		*0.02*	
	Survey (bidaily+weekly)	*3.86*		*0.05*		*0.21*		*0.02*	
**CGI^e^-severity**									
	Survey bidaily	0.84		0.01		0.03		0.03	
	Survey weekly	0.87		0.01		−0.03		0.02	
	Survey (bidaily+weekly)	0.84		0.01		0.03		0.02	
**CDS^f^ total score**									
	Survey bidaily	*2.48*		*0.03*		*0.25*		*0.02*	
	Survey weekly	*2.52*		*0.03*		*0.23*		*0.02*	
	Survey (bidaily+weekly)	*2.45*		*0.03*		*0.27*		*0.02*	

^a^RMSE: root mean square error.

^b^PANSS: Positive and Negative Syndrome Scale.

^c^Italic values indicate that the uncorrected *P* value for R^2^ is <.05 for elastic net models.

^d^BPRS: modified Brief Psychiatric Rating Scale.

^e^CGI: Clinical Global Impression

^f^CDS: Calgary Depression Scale.

**Table 10:** Performance of the elastic net models for predicting the Pittsburgh Sleep Quality Index global and component scores.

Feature set			RMSE^a^		SD		R^2^		SD
**PSQI^b^ global score**									
	Philips Actigraph sleep	2.68		0.01		–0.01		0.01	
	Philips Actigraph activity	2.67		0.01		–0.01		0.01	
	Philips Actigraph (sleep+activity)	2.67		0.01		–0.01		0.01	
	Garmin activity	2.62		0.02		0.03		0.01	
	Survey bidaily	*2.40^c^*		*0.03*		*0.19*		*0.02*	
	Survey weekly	*2.41*		*0.02*		*0.18*		*0.02*	
	Survey (bidaily+weekly)	*2.41*		*0.03*		*0.18*		*0.02*	
**PSQI subjective sleep quality**									
	Philips Actigraph sleep	0.76		0.01		–0.01		0.01	
	Philips Actigraph activity	0.75		0.00		–0.01		0.01	
	Philips Actigraph (sleep+activity)	0.76		0.00		–0.01		0.01	
	Garmin activity	0.76		0.00		–0.01		0.01	
	Survey bidaily	*0.73*		*0.01*		*0.05*		*0.02*	
	Survey weekly	*0.74*		*0.01*		*0.03*		*0.01*	
	Survey (bidaily+weekly)	*0.73*		*0.01*		*0.04*		*0.02*	
**PSQI sleep latency**									
	Philips Actigraph sleep	1.64		0.01		–0.01		0.01	
	Philips Actigraph activity	*1.58*		*0.01*		*0.05*		*0.01*	
	Philips Actigraph (sleep+activity)	*1.60*		*0.01*		*0.04*		*0.01*	
	Garmin activity	1.64		0.01		−0.01		0.01	
	Survey bidaily	1.62		0.01		0.02		0.01	
	Survey weekly	1.63		0.01		−0.00		0.01	
	Survey (bidaily+weekly)	1.61		0.01		0.02		0.01	
**PSQI sleep duration**									
	Philips Actigraph sleep	*1.43*		*0.02*		*0.13*		*0.02*	
	Philips Actigraph activity	*1.42*		*0.02*		*0.15*		*0.02*	
	Philips Actigraph (sleep+activity)	*1.44*		*0.01*		*0.12*		*0.02*	
	Garmin activity	*1.50*		*0.02*		*0.06*		*0.02*	
	Survey bidaily	*1.52*		*0.01*		*0.03*		*0.01*	
	Survey weekly	1.55		0.01		−0.01		0.01	
	Survey (bidaily+weekly)	1.52		0.01		0.03		0.01	
**PSQI habitual sleep efficiency**									
	Philips Actigraph sleep	7.38		0.02		−0.00		0.01	
	Philips Actigraph activity	7.38		0.03		−0.00		0.01	
	Philips Actigraph (sleep+activity)	7.38		0.02		−0.00		0.01	
	Garmin activity	7.38		0.02		−0.00		0.01	
	Survey bidaily	7.38		0.03		−0.00		0.01	
	Survey weekly	7.38		0.02		−0.00		0.01	
	Survey (bidaily+weekly)	7.38		0.02		−0.00		0.01	
**PSQI sleep disturbances**									
	Philips Actigraph sleep	3.76		0.08		0.05		0.04	
	Philips Actigraph activity	3.86		0.01		−0.00		0.01	
	Philips Actigraph (sleep+activity)	3.83		0.04		0.01		0.02	
	Garmin activity	3.85		0.02		0.00		0.01	
	Survey bidaily	*3.34*		*0.04*		*0.25*		*0.02*	
	Survey weekly	*3.35*		*0.03*		*0.24*		*0.01*	
	Survey (bidaily+weekly)	*3.27*		*0.04*		*0.28*		*0.02*	
**PSQI use of sleep medication**									
	Philips Actigraph sleep	1.35		0.00		−0.00		0.01	
	Philips Actigraph activity	1.35		0.01		−0.01		0.01	
	Philips Actigraph (sleep+activity)	1.35		0.01		−0.01		0.01	
	Garmin activity	1.36		0.01		−0.01		0.01	
	Survey bidaily	1.33		0.02		0.02		0.03	
	Survey weekly	1.32		0.01		0.03		0.02	
	Survey (bidaily+weekly)	1.34		0.02		0.02		0.02	
**PSQI daytime dysfunction**									
	Philips Actigraph sleep	0.96		0.01		−0.04		0.01	
	Philips Actigraph activity	0.96		0.01		−0.04		0.02	
	Philips Actigraph (sleep+activity)	0.97		0.01		−0.04		0.01	
	Garmin activity	0.97		0.01		−0.04		0.01	
	Survey bidaily	0.96		0.01		−0.04		0.02	
	Survey weekly	0.95		0.03		−0.01		0.06	
	Survey (bidaily+weekly)	0.96		0.02		−0.03		0.04	

^a^RMSE: root mean square error.

^b^PSQI: Pittsburgh Sleep Quality Index.

^c^Italic values indicate significant correlation (*P*<.05).

## Discussion

### Principal Findings

With the recent increase in the use of mobile technologies (smartphones) and remote sensing wearable devices (smartwatches) with inbuilt sensors, continuous tracking of activity, sleep, and self-reported parameters has been explored widely in various psychiatric disorders [9,12,13,17,19,30-33]. This study demonstrates that parameters that are potentially relevant to the detection of relapse, such as changes in activity and sleep [34], and self-reported symptoms can be collected using smart devices in patients with schizophrenia or schizoaffective disorder.

Several studies have shown that the vast majority of patients with schizophrenia report sleep abnormalities, which often tend to occur before the onset of illness and can predict an acute exacerbation of psychotic symptoms [35]. Individuals with schizophrenia also report significant changes in activity before a relapse episode [36,37]. Therefore, continuous sleep, activity level, and self-reported metrics provide an objective and time-stamped record of changes in behavior that may be clinically relevant and can become a part of the clinical dialog. Although there may be a potential in using remote sensing technologies to track physical activity and sleep behavior to detect relapses, patient adherence and acceptance can limit the effectiveness of these apps. Individuals with schizophrenia may often refuse to use such devices because of a lack of familiarity with the technologies, with using the device, and with the disease state (eg, paranoid behavior). In this study, high data coverage and compliance indicated the overall acceptance of all remote sensing mobile devices used. Our experience in this study suggests that the involvement of patients’ physician, good training, and clear communication related to the research goals and potential benefits and risks of the study are important factors in ensuring good compliance and engagement.

In this study, correlations among the individual clinical symptoms as well as between clinical symptoms and device measures were evaluated. The assessment of within-patient stability of the clinical scales showed that most patients’ symptoms were stable throughout the study. The correlation analysis among clinical symptoms indicated that the PANSS total score and the BPRS total score showed a strong correlation. Patient-reported survey data features were correlated with clinical symptoms.

In this study, an elastic net model was used to predict correlations between clinical assessments and the device metrics. The Philips Actigraph and the Garmin activity data showed some utility in predicting the sitting index of the YPAS. The features derived from the patient-reported survey data were useful in predicting the total score, the subjective sleep quality component, and the sleep disturbance component of the PSQI. Features derived from the Philips Actigraph and Garmin vivofit were predictive of the sleep duration component of the PSQI. The implementation of technology-based measures along with metrics-based prediction methods could be effective in instituting early warning systems for symptom decline and relapse patterns in schizophrenia.

### Limitations

With limited relapses or clinical changes, this study was not sufficiently powered to identify predictors of relapse or symptom exacerbation by comparing clinical and technological assessments. Although a higher relapse rate was expected based on historical rates in the clinic, we speculate that there may have been a recruitment bias for this study. Less stable subjects may have been less interested in participation. In addition, the frequent clinical assessments and questionnaires and the presence of the devices would have generated a heightened personal focus on relevant symptoms. Future trial designs should consider tracking recruitment bias in the design. In general, expanding the study to more patients and a longer duration would be required to develop robust models predictive of clinical change. The longitudinal stability of the patients would facilitate, however, the ability to detect relative changes and outliers when they occur. Although detecting change is facilitated by having a long-term stable baseline, patients that are unstable will inherently have more variability. Higher variability can make the detection of event outliers more difficult. Having continuous data, however, allows statistical averaging for longer periods than single-point estimates. Trends and changes in variability can be detected with streaming data, even in unstable subjects, more readily than with point estimates from clinical visits. This study demonstrates that remote sensing devices and mobile technology can be used to monitor metrics that are relevant for patients with schizophrenia. It will be important to capture the context of detected changes in future studies to determine the metrics most predictive of relapse or symptom exacerbation.

### Conclusions

In summary, relapse prediction using remote sensing technologies may aid clinicians to be cautioned in advance to detect the approaching exacerbation of symptoms and patterns of relapse in patients with schizophrenia. The operational learnings from this study provide insights to conduct future studies with remote sensing devices in this patient population to devise earlier intervention strategies.

## Appendix

Multimedia Appendix 1 Stability of the clinical scales during the study.

Multimedia Appendix 2 Performance of the elastic net models for predicting the global and individual Yale Physical Activity Survey indices.

## Abbreviations

BPRS: Brief Psychiatric Rating Scale
CDS: Calgary Depression Scale
CGI-SCH: Clinical Global Impression-Schizophrenia
DSM-5: Diagnostic and Statistical Manual of Mental Disorders, Fifth Edition
ICC: intraclass correlation coefficient
PANSS: Positive and Negative Syndrome Scale
PRO: patient-reported outcome
PSQI: Pittsburgh Sleep Quality Index
REDCap: Research Electronic Data Capture
UAB: University of Alabama, Birmingham
YPAS: Yale Physical Activity Survey
