# Supplementary material for: Clinical Utility of Wearable Sensors and Patient-Reported Surveys in Patients With Schizophrenia: Noninterventional, Observational Study
Source: JMIR Ment Health. 2021 Aug 9;8(8):e26234. doi: 10.2196/26234 (PMC8386407; doi:10.2196/26234)
Supplement: Multimedia Appendix 1 [file mental_v8i8e26234_app1.docx]

**Supplementary Table 1. Stability of clinical scales during the study***

| **Clinical Scale** | **ICC** | **Agreement** |
| --- | --- | --- |
|  |  |  |
| PANSS total score | 0.76 | excellent |
| BPRS total score | 0.70 | good |
| CGI-severity | 0.91 | excellent |
| CDS total score | 0.69 | good |
| YMRS total score | 0.65 | good |
| PSQI global score | 0.71 | good |
| PSQI subjective sleep quality | 0.56 | fair |
| PSQI sleep latency | 0.42 | fair |
| PSQI sleep duration | 0.72 | good |
| PSQI habitual sleep efficiency | 0.56 | fair |
| PSQI sleep disturbances | 0.59 | fair |
| PSQI use of sleep medication | 0.67 | good |
| PSQI daytime dysfunction | 0.68 | good |
| YPAS global index | 0.38 | poor |
| YPAS vigorous activity index | 0.31 | poor |
| YPAS leisure walking index | 0.54 | fair |
| YPAS moving index | 0.53 | fair |
| YPAS standing index | 0.37 | poor |
| YPAS sitting index | 0.27 | poor |

N=40 enrolled patients; BPRS, Modified Brief Psychiatric Rating Scale; CDS, Calgary Depression Scale for Schizophrenia; CGI, Clinical Global Impression; PANSS, Positive and Negative Syndrome Scale; PSQI, Pittsburgh Sleep Quality Index; YPAS, Yale Physical Activity Survey. ICC agreement: less than 0.4 – poor; between 0.4 and 0.59 – fair; between 0.60 and 0.74 – good; between 0.75 and 1 – excellent.

Interpretation of ICC agreement measures is based on guidelines provided in Cicchetti, Domenic V. (1994). "Guidelines, criteria, and rules of thumb for evaluating normed and standardized assessment instruments in psychology". *Psychological Assessment*. **6** (4): 284–290.
