# Supplementary material for: Clinical Utility of Wearable Sensors and Patient-Reported Surveys in Patients With Schizophrenia: Noninterventional, Observational Study
Source: JMIR Ment Health. 2021 Aug 9;8(8):e26234. doi: 10.2196/26234 (PMC8386407; doi:10.2196/26234)
Supplement: Multimedia Appendix 2 [file mental_v8i8e26234_app2.docx]

**Supplementary Table 2. Performance** **of elastic net models for predicting YPAS global and individual indexes**

| **Feature Set** | **RMSE (SD)** | **R squared (SD)** |
| --- | --- | --- |
|  |  |  |
| **YPAS global index** |  |  |
|  |  |  |
| Philips Actigraph sleep | 19.16 (0.08) | -0.00 (0.01) |
| Philips Actigraph activity | 19.15 (0.07) | -0.00 (0.01) |
| Philips Actigraph (sleep + activity) | 19.19 (0.07) | -0.01 (0.01) |
| Garmin activity | 19.01 (0.20) | 0.01 (0.02) |
| Survey bidaily | 18.89 (0.13) | 0.03 (0.01) |
| Survey weekly | 19.27 (0.10) | -0.01 (0.01) |
| Survey (bidaily + weekly) | 19.11 (0.14) | 0.00 (0.01) |
|  |  |  |
| **YPAS vigorous activity index** |  |  |
|  |  |  |
| Philips Actigraph sleep | **12.96 (0.15)** | **0.06 (0.02)** |
| Philips Actigraph activity | 13.35 (0.05) | 0.00 (0.01) |
| Philips Actigraph (sleep + activity) | 13.34 (0.04) | 0.00 (0.01) |
| Garmin activity | 13.33 (0.03) | 0.00 (0.00) |
| Survey bidaily | 13.37 (0.04) | -0.00 (0.01) |
| Survey weekly | 13.38 (0.04) | -0.00 (0.01) |
| Survey (bidaily + weekly) | 13.38 (0.04) | -0.00 (0.01) |
|  |  |  |
| **YPAS leisure walking index** |  |  |
|  |  |  |
| Philips Actigraph sleep | 10.16 (0.05) | -0.01 (0.01) |
| Philips Actigraph activity | 10.21 (0.05) | -0.02 (0.01) |
| Philips Actigraph (sleep + activity) | 10.16 (0.04) | -0.01 (0.01) |
| Garmin activity | 10.10 (0.05) | 0.00 (0.01) |
| Survey bidaily | **9.84 (0.08)** | **0.05 (0.01)** |
| Survey weekly | 10.17 (0.04) | -0.01 (0.01) |
| Survey (bidaily + weekly) | 10.03 (0.09) | 0.01 (0.02) |
|  |  |  |
| **YPAS moving index** |  |  |
|  |  |  |
| Philips Actigraph sleep | 3.36 (0.03) | -0.04 (0.02) |
| Philips Actigraph activity | 3.34 (0.02) | -0.03 (0.01) |
| Philips Actigraph (sleep + activity) | 3.34 (0.03) | -0.03 (0.02) |
| Garmin activity | 3.35 (0.04) | -0.03 (0.02) |
| Survey bidaily | 3.30 (0.06) | -0.00 (0.03) |
| Survey weekly | 3.35 (0.03) | -0.03 (0.02) |
| Survey (bidaily + weekly) | 3.33 (0.04) | -0.02 (0.02) |
|  |  |  |
| **YPAS standing index** |  |  |
|  |  |  |
| Philips Actigraph sleep | 3.29 (0.01) | 0.00 (0.01) |
| Philips Actigraph activity | 3.26 (0.03) | 0.02 (0.02) |
| Philips Actigraph (sleep + activity) | 3.28 (0.05) | 0.01 (0.03) |
| Garmin activity | 3.25 (0.04) | 0.02 (0.02) |
| Survey bidaily | 3.29 (0.01) | -0.00 (0.01) |
| Survey weekly | 3.30 (0.01) | -0.00 (0.01) |
| Survey (bidaily + weekly) | 3.29 (0.01) | -0.00 (0.01) |
|  |  |  |
| **YPAS sitting index** |  |  |
|  |  |  |
| Philips Actigraph sleep | 0.97 (0.01) | 0.02 (0.01) |
| Philips Actigraph activity | **0.93 (0.01)** | **0.11 (0.01)** |
| Philips Actigraph (sleep + activity) | **0.93 (0.01)** | **0.10 (0.02)** |
| Garmin activity | **0.97 (0.01)** | **0.03 (0.01)** |
| Survey bidaily | 0.98 (0.00) | 0.00 (0.01) |
| Survey weekly | 0.98 (0.01) | 0.00 (0.02) |
| Survey (bidaily + weekly) | 0.98 (0.00) | -0.00 (0.01) |

RMSE, Root Mean Square Error; YPAS, Yale Physical Activity Survey. Bold indicates significant correlation (P<0.05).
